# Overstocked Agricultural Produce and Emergency Supply System in the COVID-19 Pandemic: Responses from China

**DOI:** 10.3390/foods10123027

**Published:** 2021-12-06

**Authors:** Mingzhe Pu, Xi Chen, Yu Zhong

**Affiliations:** Institute of Agricultural Economics and Development, Chinese Academy of Agricultural Sciences, Beijing 100082, China; pumingzhe@caas.cn (M.P.); chenxi_alice@163.com (X.C.)

**Keywords:** COVID-19, overstocked agricultural products, food loss, food system, online data, e-commerce, China

## Abstract

The spread of COVID-19 has affected not only public health but also agriculture, raising global concerns regarding the food system. As an immediate impact of COVID-19, farmers around the globe have had difficulties with sales, resulting in large amounts of overstocked agricultural products and food loss. This further threatens the livelihood of rural, poor farmers and impacts sustainable production. To provide a better understanding of the overstocking situation after the outbreak of the pandemic, this study depicts the distribution characteristics of overstocked agricultural products in China. After analyzing a nationwide data set collected from 3482 individuals/organizations by the Chinese Agri-products Marketing Association after the outbreak of the pandemic, we found that some of the initial prevention and control measures disrupted sales channels, and in turn, caused the farmers to suffer losses. The impact was more severe in perishable products and their production areas, as well as in poverty-stricken regions. Then, we identified China’s quick and effective actions to match the supply and demand. These findings suggest that emergency responses should coordinate the relationship between emergency actions and the necessary logistics of agricultural production. To prepare for the possibility of such shock in the future, the government should take actions to clear logistics obstacles for necessary transportation, keep enhancing the fundamental infrastructure and effective mechanism of the food supply chain, and actively include innovative techniques to build a more resilient food system.

## 1. Introduction

The emergence of coronavirus disease 2019 (COVID-19) has imposed unprecedented challenges to the world economy. In the agricultural sector, the lockdown measures and movement restrictions disrupted food supply chains, raising concerns regarding food security [1,2,3,4,5]. Difficulties in selling agricultural products and highly overstocked situations had been reported around the globe [6,7,8,9]. In developing countries, such as China, India, and Ethiopia, farmers had to leave their produce in the field to rot [10,11,12,13,14,15,16]. Even in developed countries such as the United States and Canada [17,18,19,20], farmers had to dump milk, crush eggs and plow under crops [21,22]. The adverse effects of overstocked agricultural products have been analyzed in the current research. Firstly, overstocked agricultural products aggravate the contradiction between food supply and demand [23]. Restrictions on movement are reducing farmers’ ability to sell products. Agricultural produce is accumulating at farms, resulting in huge food losses [24,25,26]. On the demand side, indoor demand for food is strong under the current quarantine measures [27]. Some store shelves have been kept empty due to panic buying [28]. Secondly, overstocked agricultural products decrease the agricultural income of rural households [29]. In developing countries, a large subpopulation lives on agriculture and informal food markets [15,30,31]. Difficulties in sales directly threaten the livelihoods of rural, poor, and informal sector workers [32]. As estimated by the UN, an additional 83 million people, for a total of 132 million people, will experience hunger this year because of COVID-19 [33]. Thirdly, overstocked agricultural products undermine agricultural production capacity and sustainability [34]. Constrained by limited cash flow and financial liquidity [7], farmers lack incentives to invest in the next season and consequently reduce production [35]. This will increase market volatility and lead to more severe food insecurity shortly. Based on the above concerns, overstocked agricultural products in the pandemic have already aroused many concerns from the public and the government [36].

However, few academic research efforts tracked and analyzed the overstocked problems of agricultural products at the onset of the COVID-19 pandemic. The overstocking situation during the COVID-19 pandemic is quite different from that in the normal market. Previous research has suggested that excess supply caused by asymmetric market information or less organized production is the main reason for overstocking. During the COVID-19 pandemic, the problem has lain with demand and distribution channels [11,37]. On the other hand, the overstocking problem has revealed some shortcomings in the emergency food supply system. The current emergency system remains fragile in terms of logistics and distribution channels [38] and fails to coordinate the supply of agricultural products with overall crisis-response measures [39]. In China, many details concerning the overstocking problem during the spread of the COVID-19 have not been clarified yet. For instance, what are the distribution characteristics of overstocked agricultural products across the country? Are the impacts widespread, even in counties with different levels of pandemic risk, poverty, and e-commerce development? What can we learn from the government responses and the system recovery process?

This paper seeks to fill the gap in knowledge by shedding light on the above questions. To do so, we used online data from the Chinese Agri-products Marketing Association (CHAMA), including 3482 pieces of information regarding overstocking across the whole country from 20 February to 31 March 2020. These unique data allow us to provide a nationwide picture of the distribution characteristics of overstocked agricultural products. By combining information regarding overstocking with county-level data, we further examine the effects of pandemic risk, poverty, and e-commerce on difficulties in sales, providing in-depth detail on the impacts of the COVID-19 pandemic on the agricultural sector and emergency response system. This study contributes to the literature in three ways. First, to the best of our knowledge, it is the first work to use nationwide online data to investigate the impact of COVID-19 on overstocked problems. Most works are based on case studies in a certain city. Second, this article quantifies the effects of COVID-19 on the sales of agricultural products. It enriches current research on overstocked agricultural products by analyzing the problem in an emergency, which has seldom been discussed before. Third, our analysis provides descriptive and empirical evidence for academic research and policy practices to strengthen the emergency response system, and to find a way to consolidate the food supply chain and build a more sustainable food system in China. For other developing countries that are experiencing serious outbreaks, our research will aid in keeping food systems well-functioning and in protecting domestic agricultural production.

The rest of this paper is organized as follows. Section 2 introduces the research methodology. Section 3 reports the descriptive statistics and empirical results. Section 4 introduces the government responses to the overstocked problem and market responses. Section 5 discusses our findings. The final section concludes and derives several policy implications.

## 2. Research Methodology

This study employs both qualitative and quantitative analysis. For a holistic examination of the overstocking problem of the agricultural products in China after the COVID-19 lockdown, it was first important to understand the distribution characteristics of overstocked produce. Most available overstocking information were certain cases in a region or city without a nationwide representative. On 17 February 2020, the Ministry of Agriculture and Rural Affairs (MARA) called for industry associations and e-commerce enterprises to collect information on overstocking and promote sales. These data platforms allowed us to collect nationwide data and analyze the overstocking problem from a global perspective. Therefore, we collected our data on overstocking information from a large industrial association called the CHAMA. The data were scraped daily and provided information on food categories, overstocked amounts, prices, and locations.

Based on these details, we analyzed the distribution characteristics from perspectives of time-evolution trend, regional distribution, and category distribution. To reveal the potential economic loss behind the overstocked problem, we used the prices reported by the producers to estimate overstocked values.

To find the determinants of the overstocking magnitude, we combine the overstocked data with the data of exposure risk to the pandemic, poverty situation, and e-commerce development. Then we used multiple linear regression to analyze the impacts of pandemic risk, e-commerce development, and poverty on the overstocked quantities.

Government responses were important to help the market back to its normal levels. Governments’ interventions were reviewed based on the descriptive analysis, followed by the market rebound data to indicate the recovery performances. Following data analysis, mitigation strategies were identified by combining our observations in China and the literature.

### 2.1. Data

We chose the CHAMA as the data source for our research. The CHAMA, 18 years of experience in digitized supply chain management and supply-demand information matching, ranked first on the list of online assistant platforms recommended by the MARA. All its 945 association members are part of large-scale wholesale markets of agricultural produce, with national or regional influence. To collect overstocking information across the country, the CHAMA started a special online platform named “matching agricultural production and demand to fight against COVID-19 and assist farmers” on 20 February 2020. In parallel with the web platform, the CHAMA also used WeChat, email, and 24-h hotlines to facilitate information collection. To assist the “point-to-point” connection of producers and buyers, all collected information was open to the public and posted in real time on this platform.

A producer who was experiencing difficulty in sales needs only to fill out an online enrollment form, including the following information: the name of the organization, the category of the organization (government/association/other organization, agri-firm/cooperative, individual), contact person, phone number, location (accurate to the county level), product categories (grain and edible oil, vegetable, fruit, livestock and poultry, aquatic product and so on), product name, overstocked quantity, and expected price (optional). This form helped to provide standardized data about different foods, different organizations, and different regions. All data needed to be reviewed by CHAMA to ensure credibility and quality.

Web crawling was carried out from 20 February to 31 March 2020. The start date was when the CHAMA started running the online platform, and the end date was the amount of overstocked information decreased to zero.

To examine what factors might be correlated with the overstocking of agricultural products, we drew on information from both sample observations and the county from which they originated. We were interested in three key variables: risk of exposure to the pandemic, poverty levels, and e-commerce development.

The risk of exposure to the pandemic was measured by the cumulative number of confirmed COVID-19 cases at the city (district) level during the pandemic. These numbers were collected by Dingxiangyuan and Dr. Dingxiang and were posted on an online platform called “real-time dynamics of COVID-19 outbreak”. The cumulative confirmed cases for each city (district) were retrieved on 2 April 2020, two days after the deadline for collecting overstocking information. The time lag did not make a large difference, because the pandemic had been largely controlled by the end of March.

Poverty status was divided into three categories, poverty-stricken county, poverty-relieved county, and ordinary county, according to the official documents of the State Council Leading Group Office of Poverty Alleviation and Development (SCLGOPAD). In 2014, SCLGOPAD announced there were 832 poverty-stricken counties across the country. By the end of 2019, the total amount had decreased to 52 counties in seven provinces. These 52 counties were defined as poverty-stricken counties. The counties which succeeded in alleviating poverty during 2014–2019 were defined as poverty-relieved counties. The remaining counties were considered ordinary counties.

The development of e-commerce was measured by the number of Internet users at the end of the year in a county. These data came from the statistical yearbooks published by the governments of the provinces or the cities, or the national economic statistics bulletins of the counties.

We matched the pandemic risk, poverty alleviation, and e-commerce development to the overstocking information according to the county information. For some pieces of information for which the address could not be accurate to the county level, these three variables were set as vacancies.

Other data regarding government and market responses were from the government’s released documents and reports, the National Bureau of Statistics, a big-data information platform called National Data Service Platform of Agricultural and Rural Response to COVID-19.

### 2.2. Descriptive Statistics

The overstocking data were initially analyzed using descriptive statistics. We tracked changes in the amount of overstocking information over time. The proportion of each product was analyzed, indicating the most vulnerable product during the pandemic. Various subsamples, decomposed according to the categories and regions, are investigated to provide in-depth detail.

Then, the potential economic losses were estimated based on the online information. The overstocked quantities for each product could be calculated by conversion into compatible units. By timing the prices, the economic value of overstocked agricultural produces could be estimated accordingly.

The descriptive statistics method was also used to analyze government responses and market rebound. Several important government intervention measures were reviewed. Then the market transaction amount change compared to 2019 was calculated day by day. The evolution of these daily changes provided dynamic information on market responses.

### 2.3. Empirical Investigation

An econometric model was used to investigate the impact of risk of exposure to the pandemic, poverty levels, and e-commerce development on the overstocking amount. A multiple linear regression model was set as follows:

$$\begin{array}{l} overstocking_{i}=\alpha_{1}\cdot pandemic\_risk_{i}+\alpha_{2}\cdot poverty\_relieved_{i}\\ \;\;\;\;\;\;+\alpha_{3}\cdot poverty\_stricken_{i}+\alpha_{4}\cdot ecommerce_{i}+\varepsilon \end{array}$$

The dependent variable $overstocking_{i}$ represents the overstocking number of agricultural products that a producer i had during the pandemic. Considering the dependent variable is a continuous variable, the OLS method is applicable in this paper.

$pandemic\_risk_{i}$ represents the exposure risk to the pandemic. It is set as a categorical variable ordered from 1–7 according to the cumulative number of confirmed COVID-19 cases at the city (district) level during the pandemic. The grouping method followed the official reports. The value of $pandemic\_risk_{i}$ was set to 1 if no confirmed cases were reported; 2 if 1–9 confirmed cases were reported; 3 if 10–99 confirmed cases were reported; 4 if 100–499 confirmed cases were reported; 5 if 500–999 confirmed cases were reported; 6 if 1000–10,000 confirmed cases reported; 7 if over 10,000 confirmed cases reported.

Poverty status was represented by two dummy variables $poverty\_relieved_{i}$ and $poverty\_stricken_{i}$. The treatment group is the ordinary counties whose values of $poverty\_relieved_{i}$ and $poverty\_stricken_{i}$ were set to 0. $poverty\_relieved_{i}$ was set to 1 if a county succeeded in alleviating poverty during 2014–2019. $poverty\_stricken_{i}$ was set to 1 if a county was still in the listed 52 poverty-stricken counties released by SCLGOPAD in 2019. $ecommerce_{i}$ represented the number of Internet users at the end of the year in a county.

## 3. Immediate Impacts of COVID-19: Evidence from CHAMA Overstocking Information

By 31 March 2020, the CHAMA online platform had collected 3938 pieces of information regarding overstocked agricultural products. After clearing the duplicate information, there were 3482 valid observations with approximately 85 observations per day. All observations came from three categories of organizations: individual, agricultural companies/cooperatives, government/Association. 52.83% of the information was reported by agricultural companies and cooperatives, and 34.17% from individuals. The information from the government or association accounted for 13%. Relatively speaking, the large-scale producers are easier to be shocked by the pandemic [40].

### 3.1. Distribution of Overstocked Agricultural Products

**Figure 1:**
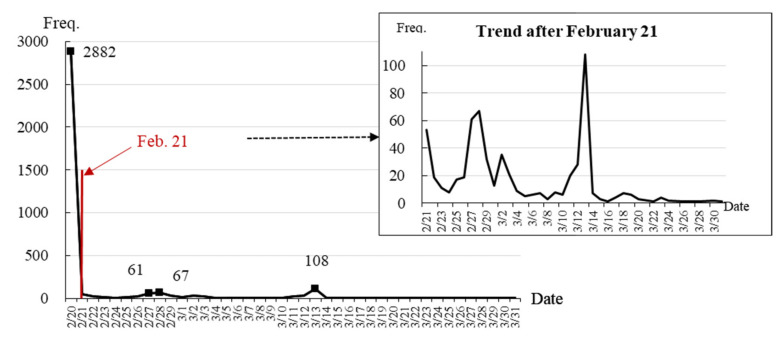
Time-evolution trend of overstocked agricultural products posted by the CHAMA from 20 February to 31 March 2020.

The time-evolution trend of overstocking information was the result of the government’s efforts to balance COVID-19 restraints and food supply chain protection. There were three peaks in the posted amount of overstocked agricultural products during the data collection period (see Figure 1). The first peak was on 20 February, when 2882 pieces of information were uploaded to the online platform. This accounted for 82.77% of the total information. The second peak was at the end of February, including 61 pieces on 27 February and 67 pieces on 28 February. The third was on 12 March, when 108 pieces of information were released. After the middle of March 2020, the amount of overstocking information decreased rapidly. The first peak was much higher than the others because the overstocking information was accumulating before the online platform was established. The national-wide lockdown measures dated back to 29 January 2020. During the 21 days before the platform was established, a large number of chicken coops, pig pens, fishponds, and farmlands were soon overstocked, and farmers could not find outflow channels. The CHAMA data showed that there was an average of 137 messages per day during this severe period. After the platform opened on 20 February, all information was reported to the platform. After 21 February, the overstocking information followed a trend of fluctuating downward (see the small figure in the upper-right corner of Figure 1). There has been no overstocked information released since April.

The pandemic struck the food supply chains across the nation. Almost all provinces (autonomous regions and municipalities) reported overstocking information of agricultural produce, especially those which produce perishable products [41]. Among 34 provinces (autonomous regions and municipalities), 32 provinces (autonomous regions and municipalities) reported overstocking cases (see Table 1). The top five provinces suffering the most from the pandemic were Guangxi, Anhui, Yunnan, Shandong, and Shanxi. Guangxi experienced the most severe overstocking situation. The amount of overstocking information in Guangxi was 2198, accounting for 63.12% of the total sample. Guangxi suffered the worst because the pandemic overlapped with its harvesting season of late-ripening citrus products. Some strict lockdown and quarantine measures cut off the traditional offline sales channels directly [32], resulting in an enormous number of overstocked produce. Anhui ranked second, with 486 pieces of information, accounting for 13.96% of the data. The proportions in Yunnan, Shandong, and Shanxi were 4.8%, 4.34%, and 4.22%, respectively. It is noted that all these provinces are the fresh “food baskets” in China, producing fruit, vegetable, livestock, and other perishable products for the rest of the country in winter. From the perspective of economic development, all these provinces were less developed. The poor farmers in these areas relied on the above cash crops to increase incomes, and the local governments to boost the rural economy. Analyzing the overstocking situation and consequent release actions is of importance not only to build a resilient food system but also for protecting the poor rural population and rural economy.

**Figure 2:**
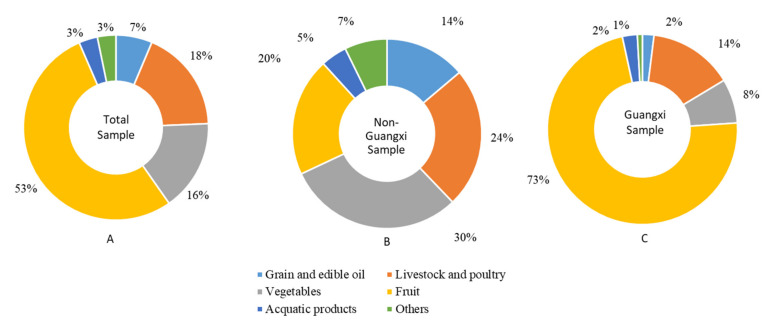
Variety distribution of overstocked agricultural products posted by the CHAMA from 20 February to 31 March 2020.

Highly perishable and time-sensitive food was highly overstocked after the pandemic. Fruit, vegetables, livestock and poultry were the top three varieties of all overstocked agricultural products. Regardless of geographic distribution, 53% of the unmarketable information was related to fruit, 18% to livestock and poultry, and 16% to vegetables. The grain and edible oil and aquatic products accounted for 7% and 3%, respectively (see Figure 2A). In the Guangxi sample (see Figure 2C), the proportion of fruit, livestock and poultry, and vegetables was 73%, 14%, and 8%, respectively. This distribution characteristic caused the proportion of fruits in the total sample to exhibit a positive bias. After excluding the Guangxi sample, the largest proportion became vegetables, accounting for 30%; the livestock and poultry still ranked second, accounting for 24%; followed by fruit, accounting for 20%. The proportion of grain and edible oil rose to 14%, and aquatic products rose to 7% (see Figure 2B). On the whole, the pandemic had a great impact on perishable agricultural products, such as vegetables, fruits, and livestock and poultry [41,42].

### 3.2. Estimation of Potential Economic Losses

The overstocked amount and economic values are directly associated with producers’ income and planting motivation for the next season. Reducing current sales not only threatened the livelihood of agricultural producers but also reduced future agricultural production. Both would bring more challenges to food security from the micro and macro levels. Although these potential risks had been identified [38], more specific analyses and calculations regarding the magnitude of agricultural produce were absent. Taking the CHAMA as a case, we could estimate the total amount (in weight) and economic value of overstocked agricultural products reported on the CHAMA online platform. Some producers provided overstocking information in the form of units of quantity. We converted all quantity units to weight units for comparison. To do so, we tried to reveal the economic burdens brought by overstocking agricultural produce during the pandemic, providing a more intuitive understanding of the potential risks behind the surface.

The total overstocked weight recorded by CHAMA was approximately 2.77 million tons (see Table 2). The largest share was fruit, reaching 1.81 million tons, followed by livestock and poultry at approximately 636.2 thousand tons. Vegetables ranked third with approximately 206.9 thousand tons. The weight of grain and edible oil and aquatic products was 73.3 thousand tons and 46.4 thousand tons, respectively. Regarding livestock and poultry, there were 21.65 million chickens, 11.08 million ducks, and 246.6 thousand pigs. From the perspective of geographic distribution, most of the overstocked grain and edible oil was located in the north-eastern region. Most other agricultural products were located in Southern China. However, after excluding the Guangxi sample, most of the overstocked fruit was located in the northern and central regions. Most unsaleable vegetables were located both in the southern and northwestern regions. Most unmarketable livestock and poultry were located in the eastern region, along with aquatic products.

A conservative estimation showed that the potential economic value of the over-stocked agricultural products (see Table 3) was approximately CNY 8.1 billion (USD 1.14 billion). On average, each organization risked losing 230 thousand CNY during the lockdowns. Livestock and poultry accounted for the largest share, reaching CNY 4.92 billion (USD 693.6 million). The second-largest share was of fruit, at approximately CNY 1.67 billion (USD 235.4 million). The economic value of overstocked grain and edible oil, vegetables, and aquatic products was approximately CNY 5 million (USD 70.5 million). The Guangxi sample still dominated the distribution of economic value. Approximately 83% of the value of livestock and poultry, and 67% of the value of fruit came from Guangxi. For other products, non-Guangxi regions contributed to over 77% for each. The above estimation showed that the breeding industry was facing the greatest financial risk during the lockdowns. Their products were not only highly perishable but also high value. If the entirety of the overstocking situation was realized, the economic losses would be too large to ignore. These losses would shock farmers’ livelihoods severely, as well as the rural economy.

Such a large number of overstocked products was not just the loss of producers and agricultural sector, but also consumers and the whole society. For the consumers, they experienced a shortage of foods due to disrupted supply chains. Panic buying and stockout drove prices high on the demand side. More consumers were in food insecurity. For the whole society, unreasonable lockdown measures resulted in a mismatch between supply and demand, causing efficiency loss to social welfare. This mismatch also led to massive food loss during the pandemic [43,44].

### 3.3. Factors Affecting the Overstocked Amount

Table 4 reports the regression results. The Variance Inflation Factor (VIF) test suggests that the explanatory variables are not strongly multicollinear. In addition, the F-statistics test shows the significant joint explanatory power of the used independent variables. The statistics validate the specifications of our models.

More overstocked agricultural products were located in cities (districts) where the pandemic was mild. This effect varied across different subgroups of products. The coefficients of grain and edible oil and livestock and poultry are negative, while one of the fruits is positive and statistically significant at the 5% level. In our data, about 73% of the overstocking samples were reported in cities (districts) where cumulative confirmed cases were between 10 and 99; 21.19 percent were reported in cities (districts) where cumulative confirmed cases were between 1 and 9. In the subgroup of fruit, the significantly positive coefficient indicates that higher exposure risk to the pandemic experienced more overstocked pressure. This confirms the previous research that the pandemic disrupted food supply chains by imposing various kinds of lockdown measures.

For other subgroups, the negative effects of exposure risk to the pandemic do not mean that difficulty in sales and overstocked agricultural products did not exist in high-risk regions. On the contrary, it was the highest pandemic risk that restricted gathering information on overstocked agricultural products, because medical treatment and rescuing life were given priority. In mild regions, the prevention and control measures were relatively loosened to resume production and boost the rural economy. Thus, the difficulties in sales in these regions could be observed more easily.

The organizations, which produce fruit in poverty-relived/poverty-stricken cities, suffer more from overstocked pressure. In the subgroup of fruit, the coefficients of poverty-stricken counties and poverty-stricken counties are significantly positive at the level of 1%. If the overstocked problem is unsolved, more poor farmers have to bear the economic losses. In the poor areas, most farmers used to plant grain for self-sufficiency due to poor conditions of transportation and less-developed markets. With infrastructure improved by the government, some poor areas tried turning to plant high-value crops such as fruit to earn more money. Many poverty-stricken households manage to shake off poverty by producing fruit. However, the logistics system is still vulnerable in these areas due to long-distance and complex geographical conditions. A systematic shock makes this weakness more salient. This result raises concerns over the performance of the governments’ poverty alleviation actions.

The coefficient in the total sample was biased to positive because of the large share of fruit. The coefficient of the poverty-stricken county regarding livestock and poultry is significantly negative. A possible explanation is that the average scale of the breeding industry in poverty-stricken counties is smaller. Most farmers breed livestock and poultry mainly for self-sufficiency. The coefficients of grain and edible oil in both types of counties are not significant, indicating the stability of the grain market during the pandemic [45]. The results concerning vegetables should not be taken into account, because there was no observation of poverty-stricken counties in the vegetable subsample.

The impact of e-commerce development varies across different subgroups of products. The coefficient in the fruit subsample is significantly negative, while others are significantly positive. The possible reason is that the dependence of these products on the Internet is different. Fruit demonstrates the feature of regionally specialized production in China and depends on the Internet to realize cross-region sales. The higher the level of e-commerce development, the more conducive a product is to achieving cross-regional production and demand information matching. Other products rely less on cross-regional e-commerce, because the supply of grain products is guaranteed by the “Cereal Bag” Provincial Governor Responsibility Mechanism, and the supply of livestock, poultry, and vegetables is guaranteed by the “food basket” Mayor Responsibility Mechanism. Grain consumption security is always the top priority in the food system. In addition, areas with more Internet users have better economic development and a higher population density, resulting in stricter pandemic prevention and control measures. This, in turn, impacts the supply of grain and edible oil, livestock and poultry, and vegetables.

## 4. Government Responses and Market Rebound

With an unprecedented scale of protection and control measures to isolate and control the COVID-19 pandemic, some restrictions had a severe impact on the sales of agricultural products [46,47,48]. Not long after the national lockdown, China witnessed difficulties in sales and the overstocking problem of agricultural products.

**Figure 3:**
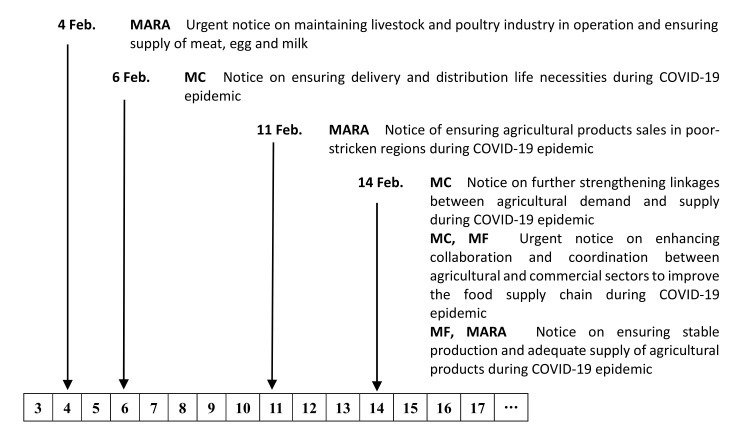
Timeline of Chinese government responses to alleviate overstocked difficulties. Notes: MARA, Ministry of Agriculture and Rural Affairs; MC, Ministry of Commerce; MF, Ministry of Finance.

Fortunately, the Chinese government has realized these problems and in February 2020 issued six urgent notices to clear logistics restrictions and to directly match production with demand [49] (see Figure 3). On February 4, the Ministry of Agriculture and Rural Affairs announced an urgent notice to maintain livestock and poultry industry operations and ensure the supply of meat, eggs, and milk. Two days later, the Ministry of Commerce (MC) announced a notice to sustain the delivery and distribution of life necessities, which included agricultural products. On February 11, the MARA announced a notice to ensure sales of agricultural products in poor-stricken regions. In February, there were three notices released by the MARA, MC, and the Ministry of Finance (MF) at the same time. The first announced actions to strengthen linkages between demand and supply of agricultural products. The second announced actions to enhance collaboration and coordination between the agricultural and commercial sectors to improve the food supply chain. The third took actions to ensure stable production and adequate supply of agricultural products.

In addition, the MARA organized industry associations and e-commerce enterprises to collect overstocked information and promote sales on 17 February 2020. As of 24 April, all major e-commerce platforms have sold 882,000 tons of fresh agricultural products and stimulated 19.8 million online transactions. The CHAMA has organized major agricultural product markets across the country to actively connect with 12 key production and marketing regions, including Hainan, Guangxi, Shandong, Hubei, and Xinjiang. A total of 2.245 million tons of agricultural products were sold by the CHAMA, reaching CNY 12.17 billion (USD 1.71 billion). Among them were 808,300 tons (CNY 4.35 billion) in Hainan, 459,500 tons in Guangxi (CNY 2.55 billion), and 281,500 tons in Shandong (CNY 1.39 billion). In addition, the MARA has launched programs to support cold chain logistics and facilitate the online selling of agricultural products.

**Figure 4:**
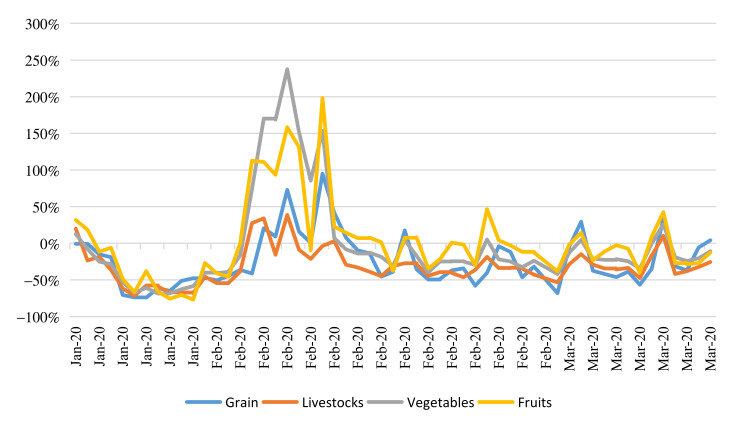
Reduction in marketing quantity of food during the COVID-19 epidemic in China compared to 2019. Note: Numbers are calculated based on a big-data information platform called National Data Service Platform of Agricultural and Rural Response to COVID-19 set by MARA.

The agricultural market rebounded soon after the government intervention. After implementation of traffic control and social distancing, the transaction number of agricultural products reduced sharply (see Figure 4). From 22 to 24 January, when grain trading was at its peak in normal years, it plunged by more than 70% compared to 2019, translating into a decline of about 30 million tons. As a result of both the epidemic and the African Swine Fever, the transaction number of livestock and poultry was 20–45% lower than the same period last year. Among them, the transaction amount on 25 January was only 13 million tons, 68.3% lower than that on 22 January, and 71 percent lower than the same period in 2019. The transaction amount recovered slightly after 22 January, but still decreased by 50–70% compared with the same period in 2019. On 9 February, the transaction amount hit the bottom again, at only 11.4 million tons. Since then, the transaction amount increased slightly and steadily, basically remaining around 20 million tons, and recovered to around 28 million tons by the end of February and mid-March. The transaction number of vegetables and fruits fell sharply first, then showed a fluctuation recovery situation. Before 10 February, the transaction number of vegetables and fruits showed a reverse trend from 2019. The trading volume of vegetables dropped from 988 million tons on 21 January to 275 million tons on 27 January, a decrease of 72.2%. Fruit trade fell 80.4% from 182 million tons to 35.8 million tons on 28th January.

## 5. Discussion

The upstream section of the food supply system suffered a lot from the COVID-19 pandemic due to inelasticity. The overstocked problems in the short term were determined by the biological nature of agricultural production [50,51]. The inevitable time lags in agricultural production weaken its ability to respond to sudden change. After the outbreak of the COVID-19, overstocked problems were observed around the globe. According to the shock transmission theory, the consequences of a shock are borne by the least elastic [44]. In our analysis, producers reported severe overstocked problems during the pandemic. The potential economic and social losses require prompt policy intervention [38].

Government intervention requires timely and accurate identification of risk points in the food supply system. A resilient food supply chain helps to enhance economic and social resilience and accelerates the recovery process. The policymakers should realize that a well-functioned food supply system is essential when coping with a sudden shock [50]. In the case of China, the government took quick lockdown actions to slow the spread of COVID-19. Fortunately, the government observed the shocks to the food supply system and released timely countermeasures. The transportation and distribution part has a vulnerability during the COVID-19 pandemic. The logistics lockdown was the key risk point. It blocked the outflow channels of agricultural products and hindered necessary production inputs and may finally destroy production cycles [51] and undermine production capacity [35]. Ensuring the minimum transportation conditions for agricultural products must be a policy priority during sudden shocks.

Innovative technologies will play a more important role in building a resilient food system [26,49]. In the case of COVID-19, the innovative technology was e-commerce. The lockdowns cut off the traditional offline channels from the producers to consumers, bringing about challenges to smooth operation of the food system. E-commerce allowed information exchange and contract transactions with minimum human-to-human interaction. A lot of retrospective research found that the individuals/organizations that were more familiar with digital tools were able to respond to and resist shocks effectively. Our empirical results also support this argument. Increasing automation and digitalization, online delivery services are changing the food supply system.

Fundamental infrastructure and effective mechanisms of the food system always matter [44]. Except for some disruption in the short term, the food supply system in China performed remarkably well during the pandemic. The policy interventions were timely in practice and the market recovered soon after the shock [35,52,53]. The remarkable performances would not have been achieved without the solid food supply basis enhanced by the Chinese government in the past decades. The “Cereal Bag” Provincial Governor Responsibility Mechanism and the “Food Basket” Mayor Responsibility Mechanism ensure the smooth operation of the food system in normal times. The national emergency food supply system consists of processing enterprises, supply outlets, distribution centers, and emergency storage and transportation enterprises to handle the emergency in abnormal times. In addition to China, the food system in Canada and the United States also worked well during the pandemic due to their resilient food system [45,50,54]. Research in India also showed that a better warehousing infrastructure can build resilience against such supply disruptions [38]. Thus, the policymakers should lay a solid foundation for the food supply system.

## 6. Conclusions

After the outbreak of COVID-19, the Chinese government has taken timely and effective prevention and control measures to contain the spread of the virus. However, some restrictions have led to difficulties in the sale of agricultural products across the country. By using 3482 pieces of overstocking information collected by the CHAMA, this study is the first to depict the distribution characteristics of overstocked agricultural products from a nationwide perspective. We seek to provide a deeper understanding of the linkage between the overstocking situation and socioeconomic development. The results suggest that the pandemic has a greater impact on perishable products. The provinces which produce fruit, vegetables, and livestock and poultry suffered the most from the shock, such as Guangxi, Anhui, Yunnan, and Shandong. Most overstocked agricultural products were located in regions where the pandemic risk was mild in February and March. The poverty-relieved and poverty-stricken counties which produce fruit faced more severe difficulties in sales. The mitigation effect of e-commerce varied across different products.

The findings of this study reveal the shortcomings in the emergency response system. It has failed to coordinate immediate actions to safeguard the necessary logistics for agricultural production. As a result, the upstream section of the food supply system suffers a lot from the COVID-19 pandemic due to its inelasticity to the market change.

The results generate new policy implications to help protect farmers and build up a more effective supply system. First, quick actions should be taken that clear the logistics bottlenecks and ensure the smooth operation of the food supply system. All unauthorized roadblocks should be prohibited. “Green channels” for agricultural products are encouraged to open across the country. Second, innovative methods should be used to enhance the resilience of the food supply system. Big data technologies can be used to share and match market information. E-commerce, as well as social media and livestreaming events, can help to promote and revive sales of agricultural products. The automation technique could provide contactless and efficient distribution services. Third, fundamental infrastructures of the food system should be consolidated. The basic elements of the food system, such as warehousing facilities, processing facilities, and logistics networks, should be strengthened at all times. Establish contingency plans for the food system for a different emergency, to make sure of timely and orderly responses.

Our study extended the work that uses online data in conjunction with other data sets for policymaking [38]. However, this study is still highly limited by the data availability. First, the large share of fruit in the CHAMA data left limited data to investigate other foods. Second, the overstocking information in the CHAMA was simple. To investigate deeper, we had to match the county-level data to the individual-level overstocking data, which lead to information loss. The simple information limited more in-depth investigation. Third, the CHAMA data is only a part of overstocked information during the pandemic. In addition to CHAMA, China Good Agri-Products Development & Service Association (CGAPA), Pinduoduo, Yimutian, Alibaba, and other organizations also established data platforms and collected information during the pandemic. Actually, the pandemic accelerated the development of e-commerce and relative online service. With more data, even the world-wide data available, a better understanding of the overstocking problem can be provided. Real-time policymaking to enhance the resilience of the food supply system can be realized.

## Figures and Tables

**Table 1:** Regional distribution of overstocked agricultural products posted by the CHAMA from 20 February to 31 March 2020.

Province	Freq.	Percent	Province	Freq.	Percent
Guangxi	2198	63.12	Heilongjiang	13	0.37
Anhui	486	13.96	Shaanxi	11	0.32
Yunnan	167	4.8	Gansu	9	0.26
Shandong	151	4.34	Sichuan	8	0.23
Shanxi	147	4.22	Qinghai	8	0.23
Hainan	65	1.87	Zhejiang	7	0.2
Jilin	33	0.95	Liaoning	7	0.2
Hebei	28	0.8	Chongqing	4	0.11
Hubei	23	0.66	Jiangsu	3	0.09
Inner Mongolia	20	0.57	Jiangxi	3	0.09
Henan	19	0.55	Fujian	3	0.09
Tianjin	17	0.49	Guangdong	2	0.06
Xinjiang	17	0.49	Shanghai	1	0.03
Hunan	17	0.49	Beijing	1	0.03
Guizhou	13	0.37	Ningxia	1	0.03
Total	3482	100			

**Table 2:** Distribution of overstocked agricultural products in different regions by CHAMA (unit: ton).

Region	Grain and Edible Oil	Fruit	Vegetables	Livestock and Poultry	Aquatic Products
North	12,824	155,537	2309	300	0
Northeast	30,365	201	460	1	20
East	15,876	5996	21,383	54,204	29,340
Central	4251	132,806	9213	140	560
South	3971	1,499,299	105,363	540,733	16,466
—excluding Guangxi	200	63,566	36,719	8	0
Southwest	2749	2885	21,660	17,757	0
Northwest	3220	10,326	46,491	23,042	0
Total	73,256	1,806,882	206,879	636,177	46,386

Notes: The North China includes Beijing, Tianjin, Hebei, Shanxi, Inner Mongolia. The Northeast region includes Jilin, Heilongjiang, and Liaoning. The East region includes Shanghai, Jiangsu, Zhejiang, Anhui, Fujian, Jiangxi and Shandong. The Central region includes Henan, Hubei, and Hunan. The South region includes Hainan, Guangdong, and Guangxi. The Southwest region includes Chongqing, Sichuan, Guizhou, Yunnan, and Tibet. The Northwest region includes Shaanxi, Gansu, Qinghai, Ningxia, and Xinjiang. There is no overstocked information reported in other provinces/regions.

**Table 3:** Estimated economic value of overstocked agricultural products (unit: million CNY).

Products		Grain and Edible Oil	Fruit	Vegetables	Livestock and Poultry	Aquatic Products
Total sample		458.95	1673.09	604.10	4924.75	462.78
Guangxi sample	value	8.93	1124.90	136.15	4108.37	78.28
	percent	2%	67%	23%	83%	17%
Non-Guangxi sample	value	450.02	548.19	467.95	816.38	384.50
	percent	98%	33%	77%	17%	83%

**Table 4:** Impacts of various factors on the overstocked amount of different agricultural products.

Variables	Total	Grain and Edible Oil	Livestock and Poultry	Vegetables	Fruit
					
Epidemic risk	−0.269**	−1.044 ***	−2.182 ***	0.186	0.361 ***
	(−2.53)	(−3.10)	(−5.16)	(0.75)	(2.77)
Poverty-relieved county	2.225 ***	−0.246	0.772	−0.456	2.443 ***
	(20.92)	(−0.51)	(1.45)	(−1.21)	(24.18)
Poverty-stricken county	0.649 ***	0.390	−2.048 **	—	0.740 ***
	(3.33)	(0.48)	(−2.42)	—	(4.45)
Internet user amount	−0.027	1.300 ***	0.608 *	0.392 **	−0.273 ***
	(−0.30)	(3.25)	(1.89)	(2.26)	(−2.74)
Western region	−0.340	0.527	0.476	0.389	−1.003*
	(−1.26)	(0.41)	(0.51)	(1.32)	(−1.65)
Central region	−1.586 ***	1.068 **	−1.777 ***	−1.040 ***	0.372
	(−11.49)	(2.37)	(−5.93)	(−4.62)	(1.48)
Constant	4.341 ***	2.523 ***	5.684 ***	2.461 ***	3.737 ***
	(17.07)	(3.44)	(5.49)	(3.95)	(12.57)
Observations	2564	107	436	356	1587
F statistics	119.4	3.983	14.61	10.23	125.3
R-squared	0.233	0.198	0.207	0.114	0.326

Notes: Robust t-statistics in parentheses, *** *p* < 0.01, ** *p* < 0.05, * *p* < 0.1. The dependent variables and the number of Internet users are logarithm form.

## Data Availability

Publicly available datasets were analyzed in this study. This data can be found here: [http://zn.agri.cn/index/dataInfo.htm?t=1] (accessed on 1 April 2020) [http://snsj.agri.cn/cockpit-index] (accessed on 15 April 2020).
